# Supplementary figures and images for: Medical Ozone Treatment Attenuates Male Reproductive Toxicity Induced by Bleomycin, Etoposide, and Cisplatin Regimen in an Experimental Animal Model
Source: Int J Mol Sci. 2025 Sep 3;26(17):8547. doi: 10.3390/ijms26178547 (PMC12428823; doi:10.3390/ijms26178547)

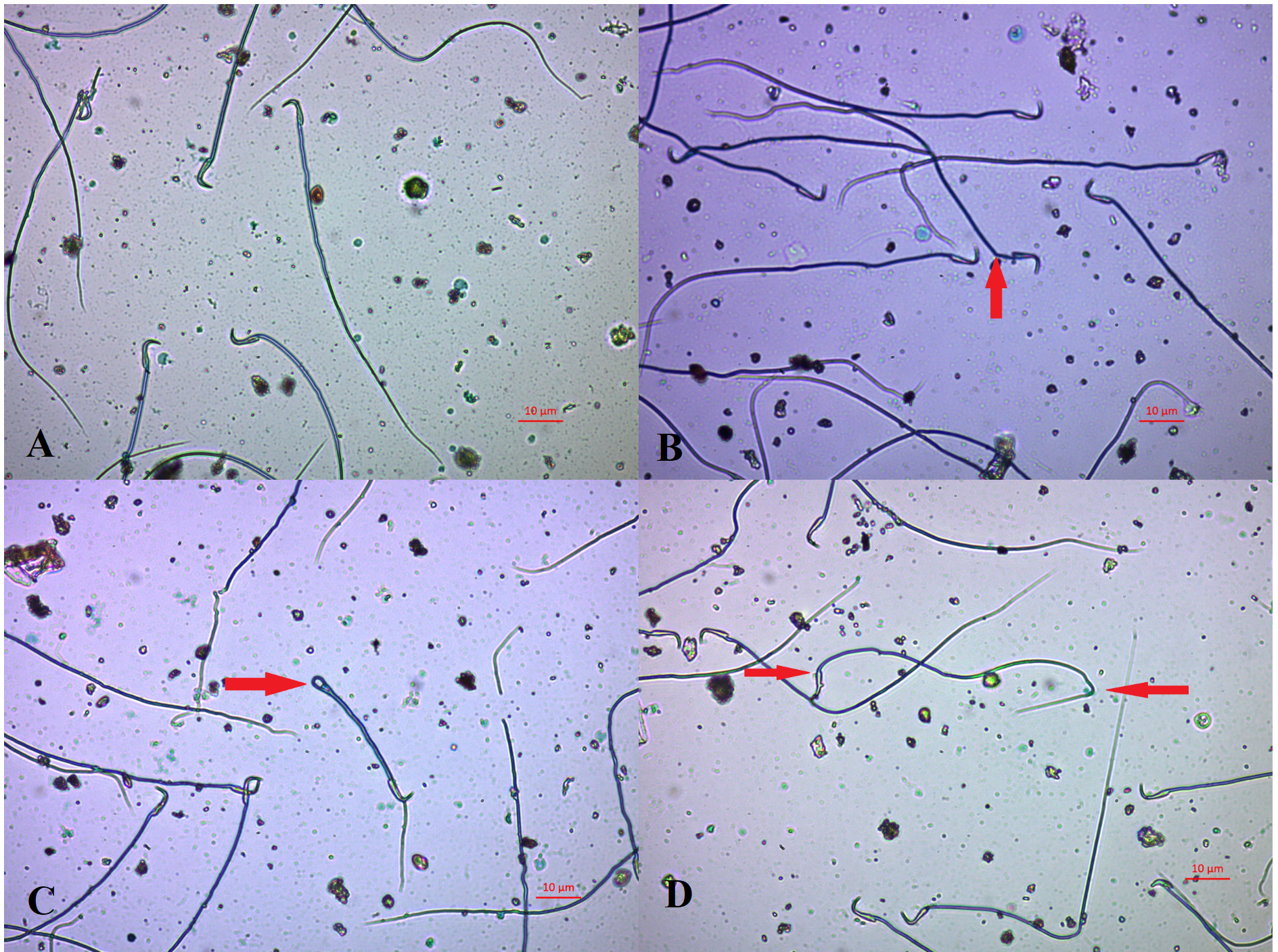

Supplement: Supplementary file 1 [file ijms-26-08547-s001.zip › Sperm morphology.png]
